# Genome Analysis of a Novel Clade II.b *Alphabaculovirus* Obtained from *Artaxa digramma*

**DOI:** 10.3390/v11100925

**Published:** 2019-10-09

**Authors:** Jiang Li, Xiaoyan Duan, Qianran Wang, Lei Zhang, Fei Deng, Hualin Wang, Zhihong Hu, Manli Wang, Jun Wang

**Affiliations:** State Key Laboratory of Virology and National Virus Resource Center, Wuhan Institute of Virology, Chinese Academy of Sciences, Wuhan 430071, China; lijiang@wh.iov.cn (J.L.); duanxiaoyan16@163.com (X.D.); wangqr0609@163.com (Q.W.); zhanglei@wh.iov.cn (L.Z.); df@wh.iov.cn (F.D.); h.wang@wh.iov.cn (H.W.); huzh@wh.iov.cn (Z.H.)

**Keywords:** ArdiNPV, baculovirus, Clade II.b *Alphabaculovirus*

## Abstract

*Artaxa digramma* is a lepidopteran pest distributed throughout southern China, Myanmar, Indonesia, and India. *Artaxa digramma* nucleopolyhedrovirus (ArdiNPV) is a specific viral pathogen of *A. digramma* and deemed as a promising biocontrol agent against the pest. In this study, the complete genome sequence of ArdiNPV was determined by deep sequencing. The genome of ArdiNPV contains a double-stranded DNA (dsDNA) of 161,734 bp in length and 39.1% G+C content. Further, 149 hypothetical open reading frames (ORFs) were predicted to encode proteins >50 amino acids in length, covering 83% of the whole genome. Among these ORFs, 38 were baculovirus core genes, 22 were lepidopteran baculovirus conserved genes, and seven were unique to ArdiNPV, respectively. No typical baculoviral homologous regions (*hr*s) were identified in the genome. ArdiNPV had five multi-copy genes including baculovirus repeated ORFs (*bro*s), calcium/sodium antiporter B (*chaB*), DNA binding protein (*dbp*), inhibitor of apoptosis protein (*iap*), and *p26*. Interestingly, phylogenetic analyses showed that ArdiNPV belonged to Clade II.b of Group II *Alphabaculoviruses*, which all contain a second copy of *dbp*. The genome of ArdiNPV was the closest to Euproctis pseudoconspersa nucleopolyhedrovirus, with 57.4% whole-genome similarity. Therefore, these results suggest that ArdiNPV is a novel baculovirus belonging to a newly identified cluster of Clade II.b *Alphabaculoviruses*.

## 1. Introduction

Baculoviruses are double-stranded DNA viruses that specifically infect the larvae of insect orders Lepidoptera, Hymenoptera and Diptera [1]. A typical baculoviral life cycle produces two distinct progeny virions: occlusion-derived virus (ODV) that initiates primary infection in the midgut epithelia of insect larvae and budded virus (BV) that spreads systemic infection within the infected larval body [2]. Baculoviruses are widely applied as biocontrol agents for pest control and as protein expression vectors [3,4,5]. According to phylogenetic analysis, *Baculoviridae* is classified into four genera: *Alphabaculovirus* (nucleopolyhedroviruses (NPVs) that specifically infect lepidopteran species), *Betabaculovirus* (granuloviruses (GVs) that specifically infect lepidopteran species), *Gammabaculovirus* (NPVs that infect hymenopteran species), and *Deltabaculovirus* (NPVs that infect dipteran species) [6,7]. Further, *Alphabaculovirus* genus can be subdivided into two large lineages (Group I and Group II) and four small lineages, while Group I contains Clade I.a and Clade I.b and Group II contains Clade II.a, Clade II.b, and Clade II.c based on the phylogenetic analysis of *late expression factor 8* (*lef-8*), *late expression factor 9* (*lef-9*), *per os infectivity factor 2* (*pif-2*), and *polyhedrin* (*polh*) genes [8].

*Artaxa digramma* (Boisduval, 1844) belongs to genus *Artaxa*, family *Lymantriidae* [9] and is distributed throughout southern China, including Jiangxi, Guangdong, and Guangxi provinces, Myanmar, Indonesia, and India. It is a pest of many plants, including pear, *Polygomum chinense*, and others [10]. In 1986, *Artaxa digramma* nucleopolyhedrovirus (ArdiNPV) was first discovered in diseased *A. digramma* in a field of Guizhou province, China [11]. In a previously reported laboratory infection assay, the mortality of ArdiNPV-infected *A. digramma* larvae was about 89% during an eight-day period [11]. The diameter of occlusion bodies (OBs) ranges from 1.09 to 2.53 μm and contains single enveloped virions.

In this study, the complete genome sequence of ArdiNPV was determined and analyzed. Phylogenetic analysis suggested that this virus is a novel Clade II.b *Alphabaculovirus*, which may be closely related to Euproctis pseudoconspersa nucleopolyhedrovirus (EupsNPV) [12].

## 2. Materials and Methods

### 2.1. Viral DNA Extraction

ArdiNPV-infected *A. digramma* larvae were preserved at the Chinese General Virus Collection Center (CGVCC) with collection number IVCAS 1.00189. Viral OBs were purified from dead larvae by differential centrifugation [13]. DNA from the viral genome was extracted using a previously described method [14].

### 2.2. Genomic DNA Sequencing and Bioinformatics Analysis

Reads of the ArdiNPV DNA were generated using the Roche 454 GS FLX pyrosequencing system. Subsequently, the reads were filtered and underwent de novo assembly into contigs using the 454 Newbler software (version 2.7). Gaps or ambiguous regions were further confirmed by PCR and Sanger sequencing. The complete genome and annotation information of ArdiNPV were submitted to GenBank (accession number: MN233792). The tool EMBOSS stretcher [15] was used to calculate the global similarity of the two sequences (ArdiNPV and EupsNPV).

The Tandem Repeats Finder [16] and BLAST [17] were employed to discover homologous regions (*hr*s). FGENESV0 [18] and ORF finder [19] were used to predict hypothetical ORFs of ArdiNPV genome, with a length at or above 50 codons and minimal overlap (less than 200 bp). Further, the BLAST algorithm was used to compare and identify hypothetical ORFs against known baculoviral proteins. Gene parity plot was performed to assess the pairwise ORF synteny between ArdiNPV and selected baculoviruses [20].

### 2.3. Phylogenetic Analysis

Thirty-eight core protein sequences [21,22] were extracted from 107 sequenced baculovirus genomes (including ArdiNPV) (Tables S2 and S3) and concatenated in the same order as that in the Autographa californica MNPV (AcMNPV) genome. Alignments were performed by ClustalW with default parameters [23]. The Maximum Likelihood method was employed to construct the phylogenetic tree using Mega7 software [24] based on the LG+G model, with 1000 bootstrap values to confirm the reliability of the tree [25]. For the alignment of ChaB and DNA binding protein (dbp), ProtTest 3.4.2 was employed to calculate the best fit model of amino acid substitution [26]. Phylogenetic trees of dbp were constructed utilizing the LG+G model and ChaB utilizing the JTT+L+G model. However, all other parameters were the same as described above.

## 3. Results and Discussion

### 3.1. Sequencing and Characterization of ArdiNPV Genome

**Figure 1:**
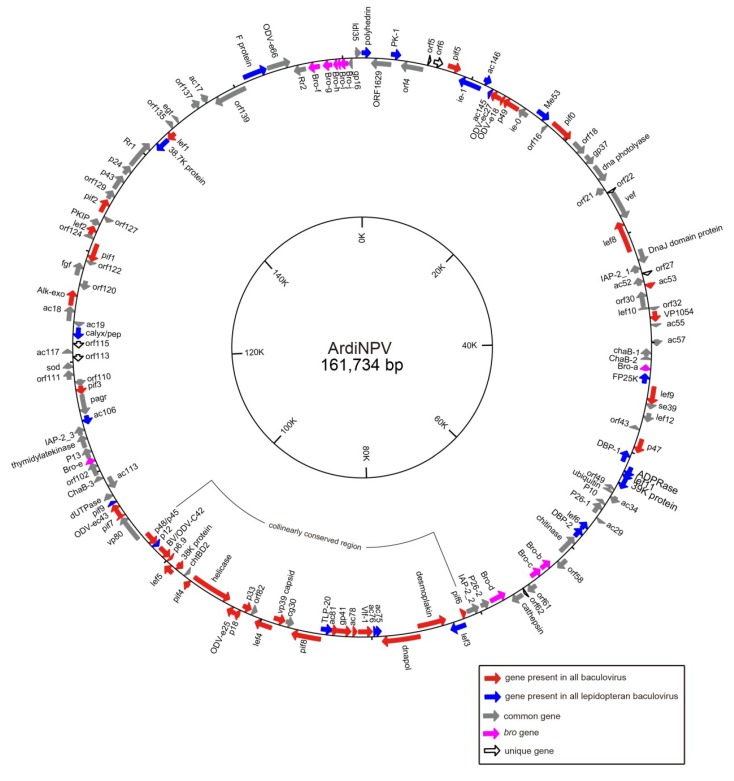
Whole-genome map of ArdiNPV. The gene transcription orientations are indicated by arrows, which depict ORFs. The gene types are colored as follows: red = core genes, blue = lepidopteran baculovirus conserved genes, gray = other baculoviral common genes, open arrows = unique genes of ArdiNPV, and purple = *bro* genes. The inner-circle indicates the gene locations. The collinearly conserved region of lepidopteran baculoviruses is also indicated.

Using the Roche 454 sequencing system, 124,744 high-quality reads of the ArdiNPV genome were generated. A complete genome of ArdiNPV was assembled using 454 Newbler software, with 230× genome coverage. The final confirmed ArdiNPV genome had a length of 161,734 bp, with 39.1% G+C content. Further, it contained 149 putative open reading frames (ORFs) beyond 50 condons, covering 83% of the ArdiNPV genome (Table S1). The *polyhedrin* gene was designated as the first ORF, and the first A of its initiation codon was defined as the start of the genome, according to the convention. In addition, 73 and 76 ORFs were in the clockwise and counterclockwise orientations, respectively, based on transcription direction of the *polyhedrin gene.* Using the BLAST algorithm, the following genes were detected in the ArdiNPV genome: 38 baculovirus core genes (red), 23 lepidopteran baculovirus conserved genes (blue), 71 other baculovirus common genes (gray), and 10 *bro* genes (purple) (Figure 1). Moreover, seven hypothetical ArdiNPV unique genes (open arrows, Figure 1) were found without homolog sequences in the NCBI database.

*Hr*s are repeated sequences with an imperfect palindromic core that is present in many baculovirus genomes. These regions act as enhancers of early gene transcription and may serve as origins of replication [27,28,29]. Although there are 4 *hr*s in EupsNPV genome, no *hr*s were found in the ArdiNPV genome. Other baculoviruses that do not contain *hr*s include Chrysodeixis chalcites NPV (ChchNPV) [30], Pseudoplusia includens NPV (PsinNPV) [31], and Trichoplusia ni SNPV (TnSNPV) of Clade II.a [32], as well as Buzura suppressaria NPV (BusuNPV) [33] and Clanis bilineata NPV (ClbiNPV) [34] of Clade II.b.

### 3.2. Phylogenetic Analysis of ArdiNPV

**Figure 2:**
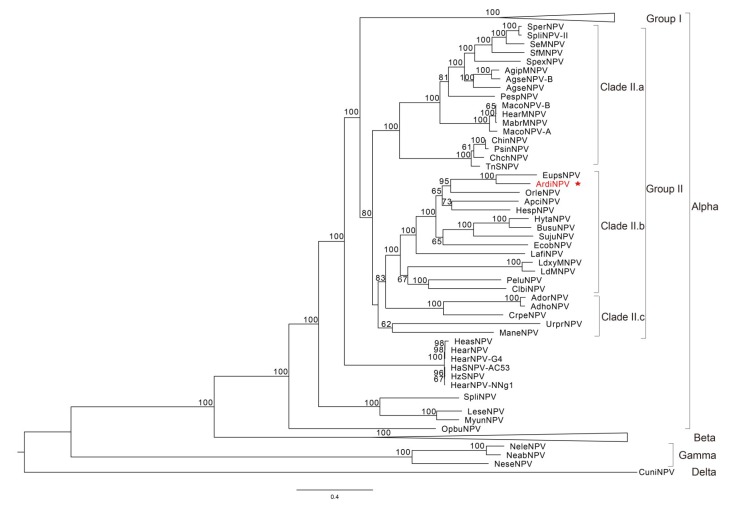
Phylogenetic tree. An unrooted tree was inferred from the concatenated alignments of 38 core genes amino acid sequences from 107 baculoviruses (Table S2) by the Maximum Likelihood method, with 1000 bootstrap values. Values of more than 50% are showed on the branch. ArdiNPV is highlighted in red.

**Figure 3:**
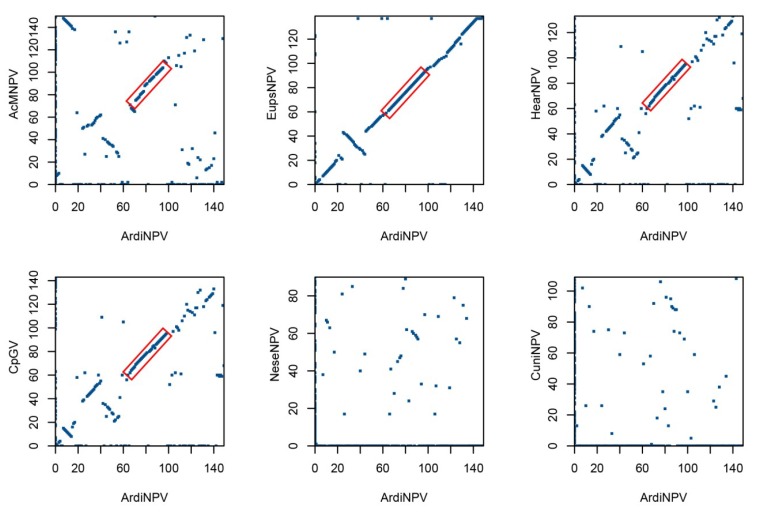
Gene parity plot analysis. Gene parity plots of ArdiNPV compared to representative baculoviruses, including AcMNPV (Group I, α), EupsNPV (Group II, α), HearNPV (alphabaculovirus minor group, α), CpGV (β), NeseNPV (γ), and CuniNPV (δ). The box indicates the lepidopteran baculovirus collinear region.

Using the Maximum Likelihood method, a phylogenetic tree was constructed based on 38 concatenated core proteins from 107 completely sequenced baculoviral genomes (including ArdiNPV), and its topological structure was largely consistent with previous studies [8]. From the phylogenetic tree, ArdiNPV (indicated by a red star) was classified as a member of Clade II.b *Alphabaculoviruses*, which forms a well-supported cluster within Clade II.b with 13 viruses, including Hemileuca sp. NPV (HespNPV) [35], Apocheima cinerarium NPV (ApciNPV), Ectropis obliqua NPV (EcobNPV) [36], Orgyia leucostigma NPV (OrleNPV) [37], Euproctis pseudoconspersa NPV (EupsNPV) [12], Sucra jujuba NPV (SujuNPV) [38], Buzura suppressaria NPV (BusuNPV) [33], Hyposidra talaca NPV (HytaNPV) [39], Lambdina fiscellaria NPV (LafiNPV) [40], Clanis bilineata NPV (ClbiNPV) [34], Perigonia lusca single NPV (PeluNPV) [41], Lymantria dispar MNPV (LdMNPV) [42] and Lymantria xylina MNPV (LyxyMNPV) [43]. Although ArdiNPV appears to be most closely related to EupsNPV (Figure 2), the whole-genome similarity between the two viruses was only 57.4% and although their genomes are mostly collinear, there is an inversion comprising genes from *orf25* (*dnaJ*) to *orf44* (*p47*) between the two genomes (Figure 3), suggesting ArdiNPV as a novel species of Clade II.b *Alphabaculoviruses*.

The ORF order and content of the ArdiNPV genome was compared with that of six representative baculoviruses, including AcMNPV (Group I), EupsNPV (Group II), Helicoverpa armigera NPV (HearNPV, alphabaculovirus minor group), Cydia pomonella GV (CpGV, betabaculovirus), Neodiprion sertifer NPV (NeseNPV, gammabaculovirus), and Culex nigripalpus NPV (CuniNPV, deltabaculovirus). ArdiNPV shared 136 homologous ORFs with EupsNPV, 116 with HearNPV, 103 with AcMNPV, 64 with CpGV, 38 with NeseNPV, and 38 with CuniNPV. For the 38 core genes, ArdiNPV shared an average amino acid (aa) identity of 64.2%, 42.3%, 42.8%, 37.1%, 31.5%, and 28.3% with the above six viruses, respectively. The gene parity plot analysis (Figure 3) showed a highly collinear gene arrangement of ArdiNPV with EupsNPV, and partial collinearity with HearNPV, AcMNPV, and CpGV. In contrast, the gene order was significantly divergent with NeseNPV and CuniNPV (Figure 3). Consistent with previous studies [33,44,45], a conserved lepidopteran baculovirus collinear region was found in ArdiNPV, which contained 20 core genes, 5 genes conserved in lepidopteran baculovirus, and 3 other baculoviral genes (Figure 1).

### 3.3. Classification of ArdiNPV Genes

Among 149 hypothetical ArdiNPV ORFs, 142 ORFs have homologs in other baculoviruses, including 15 genes potentially related to viral DNA replication, 12 to gene transcription, 31 to structure and assembly, 11 to oral infection, 27 auxiliary genes, and 46 unknown genes (Table 1). In addition, ArdiNPV was found to encode the following seven unique genes, in which, any homolog in GenBank was not found through a BLAST search: *orf5* (61 aa), *orf6* (252 aa), *orf22* (70 aa), *orf27* (95 aa), *orf62* (57 aa), *orf113* (94 aa), and *orf115* (182 aa). Further studies are required to explore whether these are functional ORFs of ArdiNPV.

### 3.4. ArdiNPV Belongs to a Cluster Clade II.b of Group II Alphabaculoviruses Which Contains a Second Copy of dbp Gene

ArdiNPV contains five multi-copy genes, including 10 copies of baculovirus repeated ORFs (*bro*s), three of calcium/sodium antiporter B *(chaB*s), two of *dbp*s, three of inhibitor of apoptosis protein *(iap*s), and two of *p26*s. So far, there have been six defined lineages of *iap* genes, named *iap*-1 to *iap*-6 in baculoviruses [46]. All the three iap genes of ArdiNPV belong to the *iap*-2 lineage, we, therefore, named them *iap*-2_1, *iap*-2_2, and *iap*-2_3. Multi-copies of genes are normally generated by gene duplication during evolution, therefore, phylogenetic analysis of those genes may provide insight into their evolutionary history. Here we focused on the phylogeny of *chaB*s and *dbp*s.

**Figure 4:**
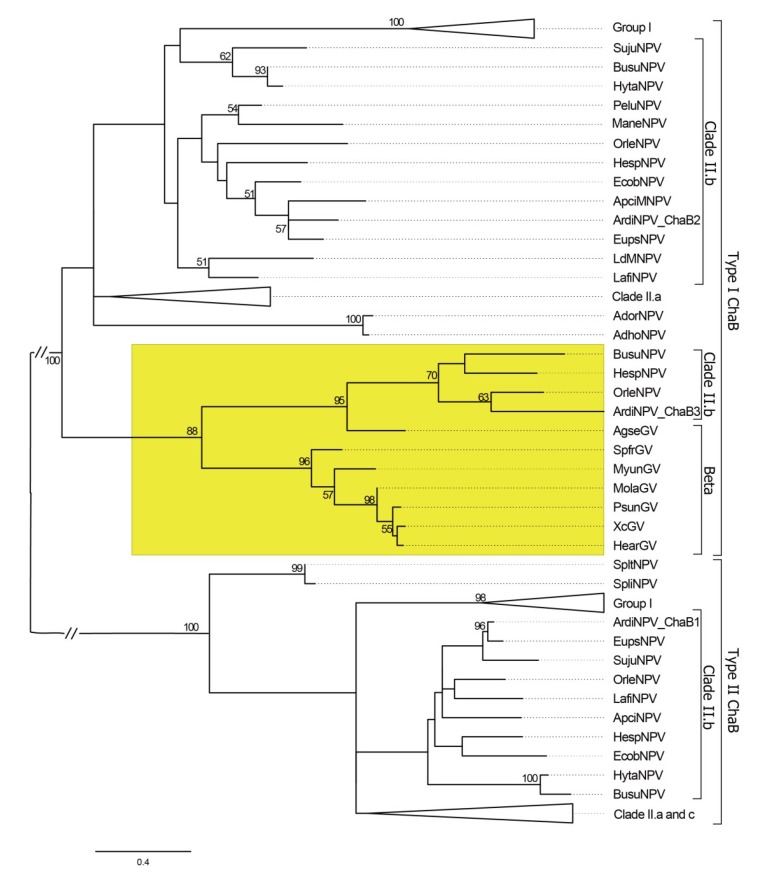
Phylogeny analysis of the baculovirus ChaB protein. The phylogenetic tree was constructed using the available baculovirus ChaB proteins by the Maximum Likelihood method with 1000 bootstrap values. Values of more than 50 are shown on the branch. The taxonomy lineages of the viruses and the types of ChaB are indicated on the right. The lineage of the third ChaB of ArdiNPV, BusuNPV, HespNPV, and OrleNPV and that of GVs is markered with a yellow box.

ChaB is conserved in all completely sequenced alphabaculovirus and also in some GVs. It is a putative DNA binding protein and contains a 60-residue conserved domain in the N-terminal region. In HearNPV, *chaB* homologous gene is involved in viral DNA replication and BV production and is transcribed in the early stage of infection [47]. Most alphabaculoviruses contain two copies of *chaB*, while ArdiNPV and three other Clade II.b viruses (BusuNPV, HespNPV, and OrleNPV) have three copies of *chaB* in their genomes. A phylogenetic tree of baculovirus ChaB was conducted and the result is shown in Figure 4. According to phylogenetic tree, the two copies of alphabaculovirus ChaB proteins (Type I and Type II) are well separated, while the third ChaB clustered with those from GVs and is grouped within Type I. Interestingly, the third ChaB of ArdiNPV, BusuNPV, HespNPV, and OrleNPV are closely related to the ChaB of Agrotis segetum GV (AgseGV) (with bootstrap value of 95%), suggesting the third ChaB may come from GVs (Figure 4).

**Figure 5:**
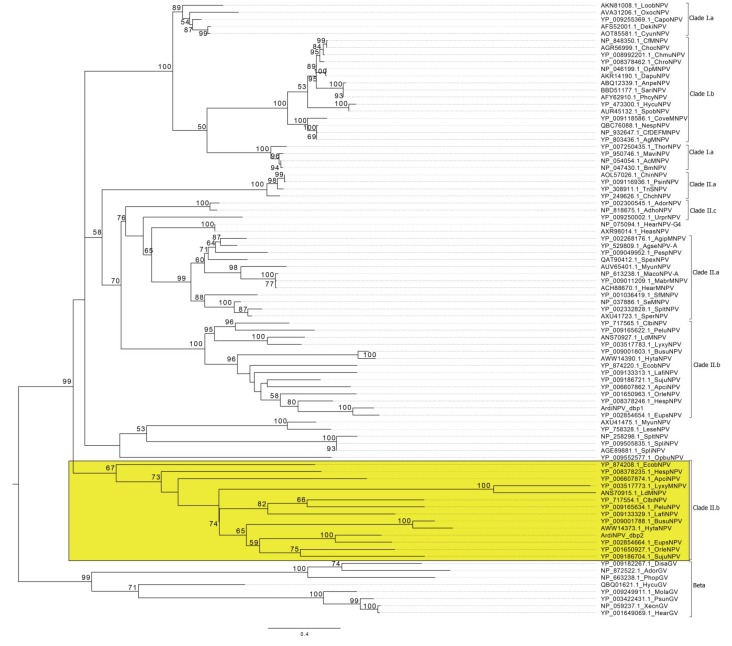
Analysis of the *dbp* duplicated gene. The phylogenetic tree was constructed using the baculovirus DBP protein by the Maximum Likelihood method with 1000 bootstrap values. Values of more than 50% are showed on the branch. The taxonomy lineages of the viruses are indicated on the right. The Clade II.b which has a second copy of *dbp* is markered with a yellow box.

The phylogenetic tree of all baculoviral DBP proteins is shown in Figure 5. To date, *dbp* has been found in all sequenced baculoviruses, except CuniNPV, and 14 baculoviruses contain a second copy of *dbp*. Interestingly, all 14 baculoviruses that contain a second copy of *dbp* belonged to Clade II.b (Figure 5). The lineage of the second copy of *dbp* (*dbp*-2) grouped well with that of *dbp*-1 of alphabaculoviruses (with bootstrap value of 99%). It is likely obtained by gene duplication of *dbp*-1 in the ancestor of these viruses and remained during their evolution. DBP can unwind short DNA strands, protect ssDNA from hydrolysis reactions, and function as an intermediate in the DNA replication process [48]. Further, the *dbp* gene is essential for BV production [49]. Although it remains unclear whether there is redundancy in the function of the second *dbp* copy, it can serve as a useful marker to distinguish viruses containing two copies of *dbp*s from other members of Group II *Alphabaculoviruses*.

## 4. Conclusions

In this study, the ArdiNPV genome was annotated and compared against other baculoviruses. Our results showed that ArdiNPV is a novel Clade II.b member which was most closely related to EupsNPV, with 57.4% genome similarity. Interestingly, among the 107 baculoviruses with full genome sequence, we found that only the members of Clade II.b contain a second copy of *dbp*, suggesting that the two copies of *dbp* can serve as a marker of the lineage. Also, some of the Clade II.b viruses contain a third copy of ChaB. Previously, the hosts of the Clade II.b have been shown to be insects specifically infecting woody plants [8], indicating there are some common genetic and ecological features of this lineage. These discoveries allowed a greater understanding of baculoviral evolution from a wider perspective.

## Figures and Tables

**Table 1:** Gene contents of ArdiNPV.

Gene Type	Core Genes	Lepidoptera Baculovirus Conserved Genes	Other Baculovirus Genes
Replication	*dna-pol(orf70), helicase(orf86), Alk-exo(ORF119), lef2(orf125), lef1(orf134)*	*ie-1 (orf8), me53 (orf15), lef11 (orf47), dbp-1 (orf45), dbp-2(orf56), lef3 (orf68)*	*dna photolyase(orf20), dUTPase(orf99), rr1(orf132), rr2(orf142)*
Transcription	*lef8(orf24), lef9(orf40), p47(orf44), vlf-1(orf73), lef4(orf81), lef5(orf90)*	*pk-1 (orf3), 39k (orf48), lef6 (orf55)*	*lef10 (orf32), lef12 (orf42),ie-0(orf14)*
Structure	*odv-ec27 (orf11), odv-e18 (orf12), 49k (orf13), ac53(orf29), vp1054(orf33), desmoplakin (orf69), ac78 (orf74), gp41 (orf75), ac81 (orf76), vp39 (orf80), p33 (orf83), p18 (orf84), odv-e25 (orf85), 38k (orf89), p6.9 (orf91), BV/ODV-C42 (orf92), p48/p45 (orf94), odv-ec43 (orf97)*	*polyhedrin (orf1), fp25k (orf39), tlp-20 (orf77), p12 (orf93), F (orf140), calyx/pep (orf116)*	*p10 (orf52), vp80 (orf95), pkip (orf126), p24 (orf131), gp16 (orf148), ORF1629 (orf2), cg30 (orf79)*
Oral infection	*pif5 (orf7), pif0 (orf17), pif6 (orf67), pif8 (orf78), pif4 (orf87), pif7 (orf96), pif3 (orf109), pif1 (orf123), pif2 (orf128)*	*pif9 (orf98)*	*odv-e66 (orf141)*
Auxiliary		*ADPRase (orf46), 38.7k (orf133)*	*iap-2_1 (orf26), ubiquitin (orf50), p26-1 (orf53), chitinase (orf57), cathepsin (orf63), gp37 (orf19), p26-2 (orf65), iap-2_2 (orf66), iap-2_3 (orf106), sod (orf112), fgf (orf121), egt(orf136), bro-a(orf38), bro-b(orf59), bro-c(orf60), bro-d(orf64), bro-e(orf103), bro-f(orf143), bro-g(orf144), bro-h(orf145), bro-i(orf146), bro-j(orf147), vef(orf23), ring finger protein(orf82), p13(orf104)*
Unknown		*ac146* (*orf9*), *ac145* (*orf10*), *ac75* (*orf71*), *ac76* (*orf72*), *ac106* (*orf107*)	*ac52 (orf28), orf31, ac55 (orf34), ac57 (orf35), chaB-1 (orf36), chaB-2 (orf37), ac34 (orf51), ac29 (orf54), chtBD2 (orf88), ac113 (orf100), ac117 (orf114), ac19 (orf117), ac18 (orf118), ac17 (orf138), DnaJ domain protein (orf25), p43(orf130), hoar(orf4), orf16, orf18, orf21, orf30, orf41, orf43, orf49, orf58, orf61, chaB-3(orf101),orf102, orf110,HE65(orf111), orf120, orf122, orf124, orf127, orf129, orf135, orf137, ldl35(orf149), thymidylate kinase(orf105), pagr(orf108), peptidase MA superfamily(orf139)*

## Supplementary Materials

The following are available online at https://www.mdpi.com/1999-4915/11/10/925/s1, Table S1: genome annotation, Table S2: Basic information of all sequenced baculovirus genomes in GenBank, Table S3: core genes of 107 genome.
