# Supplementary material for: Genome Analysis of a Novel Clade II.b Alphabaculovirus Obtained from Artaxa digramma
Source: Viruses. 2019 Oct 9;11(10):925. doi: 10.3390/v11100925 (PMC6832367; doi:10.3390/v11100925)
Supplement: Supplementary file 1 [file viruses-11-00925-s001.zip › viruses-580147-for conversion-supplementary/Supporting Table2_Basic information of all sequenced baculovirus genomes in GenBank.pdf]

S2 Table. Basic information of all sequenced baculovirus genomes in GenBank*.

Genus	Virus	Strain/isolate	Abbreviation	Accession no.	Length (bp)	Orfs	*bros*	G+C (%)	Reference
Alphabaculovirus (Group I)	Antheraea pernyi NPV	Liaoning	AnpeNPV	NC_008035	126629	147	2	53.5	[1]
	Antheraea pernyi NPV	Liaoning	AnprNPV	LC194889	126593	150	2	53.3	NP
	Antherea yammamai NPV	Nagano	AnyaNPV	LC375537	126270	152	2	53.3	NP
	Anticarsia gemmatalis NPV	AgMNPV-2D	AgMNPV	NC_008520	132239	158	7	44.5	[2]
	Autographa californica MNPV	C6	AcMNPV	NC_001623	133894	156	1	40.7	[3]
	Bombyx mandarina NPV	T3	BomaNPV	NC_012672	126770	141	3	40.2	[4]
	Bombyx mori NPV	T3	BmNPV	NC_001962	128413	143	5	40.4	[5]
	Catopsilia Pomona NPV	416	CapoNPV	KU565883	128058	131	1	39.7	[6]
	Choristoneura fumiferana DEF MNPV	-	CfDEFMNPV	NC_005137	131160	149	4	45.8	[7]
	Choristoneura fumiferana MNPV	-	CfMNPV	NC_004778	129593	146	1	50.1	[8]
	Choristoneura murinana NPV	Darmstadt	ChmuNPV	NC_023177	124688	147	1	50	[9]
	Choristoneura occidentalis NPV	BC1	ChocNPV	NC_021925	128446	148	2	50.1	[10]
	Choristoneura rosaceana NPV	NB_1	ChroNPV	NC_021924	129052	149	2	48.6	[10]
	Condylorrhiza vestigialis MNPV	-	CoveMNPV	NC_026430	125767	138	9	42.9	[11]
	Cyclophragma undans NPV	Whiov	CyunNPV	KT957089	142900	147	10	45	NP
	Dasychira pudibunda NPV	ML1	DapuNPV	KP747440	136761	161	3	54.4	[12]
	Dendrolimus kikuchii NPV	YN	DekiNPV	JX193905	141454	147	9	47.8	NP
	Epiphyas postvittana NPV	-	EppoNPV	NC_003083	118584	136	1	40.7	[13]
	Hyphantria cunea NPV	-	HycuNPV	NC_007767	132959	148	5	45.5	[14]
	Lonomia obliqua MNPV	SP/2000	LoobNPV	KP763670	120023	134	1	35.7	NP
	Maruca vitrata MNPV	-	MaviMNPV	NC_008725	111953	126	0	38.6	[15]
	Neophasia sp. alphabaculovirus	11	NespNPV	MK293724	129018	144	6	44.6	NP
	Orgyia pseudotsugata MNPV	-	OpMNPV	NC_001875	131995	152	0	55	[16]
	Oxyplax ochracea NPV	435	OxocNPV	MF143631	113971	124	0	31.1	[17]
	Philosamia cynthia ricini NPV	GX-1	PhcyNPV	JX404026	125376	138	2	53.7	[18]
	Plutella xylostellaMNPV	C6	PlxyMNPV	NC_008349	134417	152	2	40.7	[19]
	Rachiplusia ou MNPV	C6	RoMNPV	NC_004323	131526	149	0	39.1	[20]
	Samia Cynthia NPV	Nagano	SacyNPV	LC375538	126094	152	2	53.3	NP
	Samia ricini nucleopolyhedrovirus	Guangxi	SariNPV	LC375541	125921	149	2	53.5	NP
	Spilosoma obliqua NPV	IIPR	SpobNPV	KY550224	136141	135	6	45.4	NP
	Thysanoplusia orichalcea NPV	P2	ThorNPV	NC_019945	132978	145	2	39.5	[21]

Alphabaculovirus (Group II and other alphabaculovirus)	Adoxophyes honmai NPV	ADN001	AdhoNPV	NC_004690	113220	125	4	35.6	[22]
	Adoxophyes orana NPV	English	AdorNPV	NC_011423	111724	121	3	35	[23]
	Agrotis ipsilon MNPV	Illinois	AgipMNPV	NC_011345	155122	163	5	48.6	[24]
	Agrotis segetum NPV	A	AgseNPV-A	NC_007921	147544	153	4	45.7	[25]
	Agrotis segetum NPV B	B	AgseNPV-B	NC_025960	148981	150	2	45.7	[26]
	Apocheima cinerarium NPV	-	ApciNPV	NC_018504	123876	118	1	45	NP
	Buzura suppressaria NPV	Hubei	BusuNPV	NC_023442	120420	127	3	36.8	[27]
	Chrysodeixis chalcites NPV	-	ChchNPV	NC_007151	149622	151	4	39	[28]
	Chrysodeixis includens NPV	IA	ChinNPV	KU669289	140808	142	3	39.2	[29]
	Clanis bilineata NPV	DZ1	ClbiNPV	NC_008293	135454	129	3	37.7	[30]
	Cryptophlebia peltastica NPV	SA	CrpeNPV	MH394321	115728	126	3	37.2	[31]
	Ectropis obliqua NPV	A1	EcobNPV	NC_008586	131204	126	2	37.6	[32]
	Artaxa digramma NPV	424	ArdiNPV	MN233792	161734	149	10	39.1	This paper
	Euproctis pseudoconspersa NPV	Hangzhou	EupsNPV	NC_012639	141291	139	2	40.3	[33]
	Helicoverpa armigeraSNPV AC53	AC53	HaSNPV-AC53	NC_024688	130442	138	3	39.1	[34]
	Helicoverpa armigera MNPV	B	HearMNPV	NC_011615	154196	162	6	40.1	[35]
	Helicoverpa armigera NPV	C1	HearNPV	NC_003094	130759	137	3	38.9	[36]
	Helicoverpa armigera NPV G4	G4	HearNPV-G4	NC_002654	131405	135	3	39	[37]
	Helicoverpa armigera NPV NNg1	NNg1	HearNPV-NNg1	NC_011354	132425	143	4	39.2	[38]
	Helicoverpa assulta nucleopolyhedrovirus	HasNPV-DJ0031	HeasNPV	MG569706	129801	128	2	38.8	NP
	Helicoverpa zea SNPV	-	HzSNPV	NC_003349	130869	139	3	39.1	[39]
	Hemileuca sp. NPV	-	HespNPV	NC_021923	140633	137	2	38.1	[40]
	Hyposidra talaca NPV	HytaNPVIndia001	HytaNPV	MH261376	139089	141	4	39.6	[41]
	Lambdina fiscellaria NPV	GR15	LafiNPV	NC_026922	157977	137	0	43.7	[42]
	Leucania separata NPV	AH1	LeseNPV	NC_008348	168041	169	10	48.6	[43]
	Lymantria dispar MNPV	-	LdMNPV	NC_001973	161046	164	16	57.5	[44]
	Lymantria xylina MNPV	LyxyMNPV-5	LyxyMNPV	NC_013953	156344	157	14	53.5	[45]
	Malacosoma Neustria NPV	ManeNPV-T2	ManeNPV	NC_040606	130202	131	2	38.2	[46]
	Mamestra brassicae MNPV	K1	MabrMNPV	NC_023681	152710	159	6	39.9	[47]
	Mamestra configurata NPV-A	A	MacoNPV-A	NC_003529	155060	169	8	41.7	[48]
	Mamestra configurata NPV-B	B	MacoNPV-B	NC_004117	158482	168	7	40	[49]
	Mythimna unipuncta NPV	#7	MyunNPV	MF375894	148482	159	7	48.58	[50]

	Orgyia leucostigma NPV	CFS-77	OrleNPV	NC_010276	156179	135	5	39.9	[51]
	Operophtera brumata nucleopolyhedrovirus	OpbuNPV-MA	OpbuNPV	NC_040621	119054	130	2	39.8	[52]
	Peridroma NPV	GR_167	PespNPV	NC_024625	151109	139	6	53.2	[53]
	Perigonia lusca single NPV	-	PeluSNPV	NC_027923	132831	145	1	40	[54]
	Pseudoplusia includens SNPV	IE	PsinSNPV	NC_026268	139132	141	2	39.3	[55]
	Spodoptera exigua MNPV	-	SeMNPV	NC_002169	135611	139	0	43.8	[56]
	Spodoptera frugiperda MNPV virus	3AP2	SfMNPV	NC_009011	131331	143	1	40.2	[57]
	Spodoptera eridania NPV	251	SperNPV	MH320559	149090	146	2	45	NP
	Spodoptera exempta NPV	244.1	SpexNPV	MH717816	129528	139	3	41.2	NP
	Spodoptera littoralis MNPV	AN1956	SpliNPV	JX454574	137998	132	1	44.7	[58]
	Spodoptera litura NPV	G2	SpltNPV	NC_003102	139342	141	2	42.8	[59]
	Spodoptera litura NPV II	II	SpltNPV-II	NC_011616	148634	147	2	45	NP
	Sucra jujuba NPV	473	SujuNPV	KJ676450	135952	131	4	38.7	[60]
	Trichoplusia ni SNPV	-	TnSNPV	NC_007383	134394	145	2	39	[61]
	Urbanus proteus NPV	Southern Brazil	UrprNPV	NC_029997	105555	119	7	34.7	NP
Betabaculovirus	Adoxophyes orana GV	-	AdorGV	NC_005038	99657	119	0	34.5	[62]
	Agrotis segetum GV	China: Urumuqi, Xinjiang	AgseGV	NC_005839	131680	132	0	37.3	NP
	Choristoneura occidentalis GV	-	ChocGV	NC_008168	104710	116	0	32.7	[63]
	Clostera anachoreta GV	ClanGV-HBHN	ClanGV	NC_015398	101487	123	0	44.4	[64]
	Clostera anastomosis GV	HENAN	ClasGV-A	NC_022646	101818	122	0	46.7	[65]
	Clostera anastomosis GV-B	ClasGV-B	ClasGV-B	KR091910	107439	123	0	37.8	[66]
	Cnaphalocrocis medinalis GV	-	CnmeGV	KP658210	112060	101	2	35.2	[67]
	Cydia pomonella GV	Mexican 1	CpGV	NC_002816	123500	143	1	45.3	[68]
	Cryptophlebia leucotreta GV	CV3	CrleGV	NC_005068	110907	128	0	32.4	[69]
	Diatraea saccharalis GV	Parana-2009	DisaGV	NC_028491	98392	125	0	35	[70]
	Epinotia aporema GV	-	EpapGV	NC_018875	119082	132	0	41.5	[71]
	Erinnyis ello GV	S86	ErelGV	NC_025257	102759	130	0	38.7	[72]
	Helicoverpa armigera GV	-	HearGV	NC_010240	169794	179	10	40.8	[73]
	Hyphantria cunea GV	Hc1	HycuGV	MH923363	114825	132	2	39.3	NP
	Mocis latipes GV	Southern Brazil	MolaGV	KR011718	134272	145	2	38.1	NP
	Mythimna unipuncta GV	MyunGV#8	MyunGV	NC_033780	144673	153	5	49.9	[74]
	Phthorimaea operculella GV	-	PhopGV	NC_004062	119217	130	1	35.7	[75]

	Plutella xylostella GV	K1	PlxyGV	NC_002593	100999	120	0	40.7	[76]
	Plodia interpunctella GV	Cambridge	PlinGV	NC_032255	112536	123	0	44.1	NP
	Pieris rapae GV	Wuhan	PrGV	NC_013797	108592	120	0	33.2	[77]
	Pseudaletia unipuncta GV	Hawaiin	PsunGV	NC_013772	176677	183	11	39.8	NP
	Spodoptera frugiperda GV	VG008	SpfrGV	NC_026511	140913	146	7	46.2	[78]
	Spodoptera litura GV	SlGV-K1	SpliGV	NC_009503	124121	136	6	38.8	[79]
	Trichoplusia ni GV	LBIV-12	TrniGV	KU752557	175360	172	11	39.7	NP
	Xestia c-nigrum GV	-	XcGV	NC_002331	178733	181	7	40.7	[80]
Gammabaculovirus	Neodiprion abietis NPV	-	NeabNPV	NC_008252	84264	93	0	33.4	[81]
	Neodiprion lecontei NPV	-	NeleNPV	NC_005906	81755	89	0	33.3	[82]
	Neodiprion sertifer NPV	-	NeseNPV	NC_005905	86462	90	0	33.8	[83]
Deltabaculovirus	Culex nigripalpus NPV	Florida1997	CuniNPV	NC_003084	108252	109	5	50.9	[84]

*Updated by Aug 21th, 2019. NP： Unpublished.

Reference

1. Nie, Z.M.; Zhang, Z.F.; Wang, D.; He, P.A.; Jiang, C.Y.; Song, L.; Chen, F.; Xu, J.; Yang, L.; Yu, L.L., et al. Complete sequence and organization of *Antheraea pernyi* nucleopolyhedrovirus, a dr-rich baculovirus. *BMC Genomics* **2007**, *8*, 248-252, doi:10.1186/1471-2164-8-248.

2. Oliveira, J.V.d.C.; Wolff, J.L.C.; Garcia-Maruniak, A.; Ribeiro, B.M.; de Castro, M.E.B.; de Souza, M.L.; Moscardi, F.; Maruniak, J.E.; Zanotto, P.M.d.A. Genome of the most widely used viral biopesticide: *Anticarsia gemmatalis* multiple nucleopolyhedrovirus. *J. Gen. Virol.* **2006**, *87*, 3233-3250, doi:10.1099/vir.0.82161-0.

3. Ayres MD, H.S., Kuzio J, Lopez-Ferber M, Possee RD. The complete DNA sequence of *Autographa californica* nuclear polyhedrosis virus. *Virology* **1994**, *202*, 586-605.

4. Xu, Y.P.; Ye, Z.P.; Niu, C.Y.; Bao, Y.Y.; Wang, W.B.; Shen, W.D.; Zhang, C.X. Comparative analysis of the genomes of *Bombyx mandarina* and *Bombyx mori* nucleopolyhedroviruses. *J. Microbiol.* **2010**, *48*, 102-110, doi:10.1007/s12275-009-0197-4.

5. Gomi S, M.K., Maeda S. Sequence analysis of the genome of Bombyx mori nucleopolyhedrovirus. *J. Gen. Virol.* **1999**, *80*, 1323-1337.

6. Wang, J.; Zhu, Z.; Zhang, L.; Hou, D.; Wang, M.; Arif, B.; Kou, Z.; Wang, H.; Deng, F.; Hu, Z. Genome sequencing and analysis of *Catopsilia pomona* nucleopolyhedrovirus: A distinct Species in Group I Alphabaculovirus. *PLoS One* **2016**, *11*, e0155134, doi:10.1371/journal.pone.0155134.

7. Lauzon, H.A.; Jamieson, P.B.; Krell, P.J.; Arif, B.M. Gene organization and sequencing of the *Choristoneura fumiferana* defective nucleopolyhedrovirus genome. *J. Gen. Virol.* **2005**, *86*, 945-961, doi:10.1099/vir.0.80489-0.

8. de Jong, J.G.; Lauzon, H.A.; Dominy, C.; Poloumienko, A.; Carstens, E.B.; Arif, B.M.; Krell, P.J. Analysis of the *Choristoneura fumiferana* nucleopolyhedrovirus genome. *J. Gen. Virol.* **2005**, *86*, 929-943, doi:10.1099/vir.0.80490-0.

9. Rohrmann, G.F.; Erlandson, M.A.; Theilmann, D.A. Genome Sequence of an Alphabaculovirus Isolated from *Choristoneura murinana*. *Genome Announc* **2014**, *2*, e01135-01113, doi:10.1128/genomeA.01135-13.

10. Thumbi, D.K.; Beliveau, C.; Cusson, M.; Lapointe, R.; Lucarotti, C.J. Comparative genome sequence analysis of *Choristoneura occidentalis* Freeman and *C. rosaceana* Harris (Lepidoptera: Tortricidae) alphabaculoviruses. *PLoS One* **2013**, *8*, e68968,

doi:10.1371/journal.pone.0068968.

11. Castro, M.E.; Ribeiro, Z.M.; Santos, A.C.; Souza, M.L.; Machado, E.B.; Sousa, N.J.; Moscardi, F. Identification of a new nucleopolyhedrovirus from naturally-infected Condylorrhiza vestigialis (Guenee) (Lepidoptera: Crambidae) larvae on poplar plantations in South Brazil. *J. Invertebr. Pathol.* **2009**, *102*, 149-154, doi:10.1016/j.jip.2009.07.011.

12. Krejmer, M.; Skrzecz, I.; Wasag, B.; Szewczyk, B.; Rabalski, L. The genome of *Dasychira pudibunda* nucleopolyhedrovirus (DapuNPV) reveals novel genetic connection between baculoviruses infecting moths of the *Lymantriidae* family. *BMC Genomics* **2015**, *16*, 759-772, doi:10.1186/s12864-015-1963-9.

13. Hyink O, D.R., Olsen MJ, Caradoc-Davies KMB, Drake K, Herniou EA, et al. . Whole genome analysis of the Epiphyas postvittana nucleopolyhedrovirus. J. Gen. Virol. **2002**, 83, 957-971, doi:10.1099/0022-1317-83-4-957.

14. Ikeda, M.; Shikata, M.; Shirata, N.; Chaeychomsri, S.; Kobayashi, M. Gene organization and complete sequence of the Hyphantria cunea nucleopolyhedrovirus genome. J. Gen. Virol. **2006**, 87, 2549-2562, doi:10.1099/vir.0.81930-0.

15. Chen, Y.R.; Wu, C.Y.; Lee, S.T.; Wu, Y.J.; Lo, C.F.; Tsai, M.F.; Wang, C.H. Genomic and host range studies of Maruca vitrata nucleopolyhedrovirus. J. Gen. Virol. **2008**, 89, 2315-2330, doi:10.1099/vir.0.2008/001412-0.

16. Ahrens CH, R.R., Funk CJ, Evans JT, Harwood SH, Rohrmann GF. The sequence of the Orgyia pseudotsugata multinucleocapsid nuclear polyhedrosis virus genome. Virology **1997**, 229, 381-399.

17. Wang, J.; Hou, D.; Wang, Q.; Kuang, W.; Zhang, L.; Li, J.; Shen, S.; Deng, F.; Wang, H.; Hu, Z., et al. Genome analysis of a novel Group I alphabaculovirus obtained from Oxyplax ochracea. PLoS One **2018**, 13, e0192279, doi:10.1371/journal.pone.0192279.

18. Qian H, Z.Y., Wu Y, Sun P, Zhu S, et al. Analysis of the genomic sequence of Philosamia cynthia nucleopolyhedrin virus and comparison with Antheraea pernyi nucleopolyhedrin virus. BMC Genomics **2013**, 14, 115-121.

19. Harrison, R.L.; Lynn, D.E. Genomic sequence analysis of a nucleopolyhedrovirus isolated from the diamondback moth, Plutella xylostella. Virus Genes **2007**, 35, 857-873, doi:10.1007/s11262-007-0136-6.

20. Harrison, R.L.; Bonning, B.C. Comparative analysis of the genomes of Rachiplusia ou and Autographa californica multiple nucleopolyhedroviruses. J. Gen. Virol. **2003**, 84, 1827-1842,

doi:10.1099/vir.0.19146-0.

21. Wang, Y.S.; Huang, G.H.; Cheng, X.H.; Wang, X.; Garretson, T.A.; Dai, L.Y.; Zhang, C.X.; Cheng, X.W. Genome of Thysanoplusia orichalcea multiple nucleopolyhedrovirus lacks the superoxide dismutase gene. J. Virol. **2012**, 86, 11948-11949, doi:10.1128/JVI.02119-12.

22. Nakai, M. Genome sequence and organization of a nucleopolyhedrovirus isolated from the smaller tea tortrix, Adoxophyes honmai. Virology **2003**, 10.1016/s0042-6822(03)00599-3, 171-183, doi:10.1016/s0042-6822(03)00599-3.

23. Hilton, S.; Winstanley, D. Genomic sequence and biological characterization of a nucleopolyhedrovirus isolated from the summer fruit tortrix, Adoxophyes orana. J. Gen. Virol. **2008**, 89, 2898-2908, doi:10.1099/vir.0.2008/002881-0.

24. Harrison, R.L. Genomic sequence analysis of the Illinois strain of the Agrotis ipsilon multiple nucleopolyhedrovirus. Virus Genes **2009**, 38, 155-170, doi:10.1007/s11262-008-0297-y.

25. Jakubowska, A.K.; Peters, S.A.; Ziemnicka, J.; Vlak, J.M.; van Oers, M.M. Genome sequence of an enhancin gene-rich nucleopolyhedrovirus (NPV) from Agrotis segetum: collinearity with Spodoptera exigua multiple NPV. J. Gen. Virol. **2006**, 87, 537-551, doi:10.1099/vir.0.81461-0.

26. Wennmann, J.T.; Gueli Alletti, G.; Jehle, J.A. The genome sequence of Agrotis segetum nucleopolyhedrovirus B (AgseNPV-B) reveals a new baculovirus species within the Agrotis baculovirus complex. Virus Genes **2015**, 50, 260-276, doi:10.1007/s11262-014-1148-7.

27. Zhu, Z.; Yin, F.; Liu, X.; Hou, D.; Wang, J.; Zhang, L.; Arif, B.; Wang, H.; Deng, F.; Hu, Z. Genome sequence and analysis of Buzura suppressaria nucleopolyhedrovirus: a group II Alphabaculovirus. PLoS One **2014**, 9, e86450, doi:10.1371/journal.pone.0086450.

28. van Oers, M.M.; Abma-Henkens, M.H.; Herniou, E.A.; de Groot, J.C.; Peters, S.; Vlak, J.M. Genome sequence of Chrysodeixis chalcites nucleopolyhedrovirus, a baculovirus with two DNA photolyase genes. J. Gen. Virol. **2005**, 86, 2069-2080, doi:10.1099/vir.0.80964-0.

29. Craveiro, S.R.; Santos, L.A.; Togawa, R.C.; Inglis, P.W.; Grynberg, P.; Ribeiro, Z.M.; Ribeiro, B.M.; Castro, M.E. Complete genome sequences of six Chrysodeixis includens nucleopolyhedrovirus isolates from Brazil and Guatemala. Genome Announc **2016**, 4, e01192-01116, doi:10.1128/genomeA.01192-16.

30. Zhu, S.Y.; Yi, J.P.; Shen, W.D.; Wang, L.Q.; He, H.G.; Wang, Y.; Li, B.; Wang, W.B. Genomic sequence, organization and characteristics of a new nucleopolyhedrovirus isolated from Clanis bilineata larva. BMC Genomics **2009**, 10, 91-100, doi:10.1186/1471-2164-10-91.

31. Marsberg, T.; Jukes, M.D.; Krejmer-Rabalska, M.; Rabalski, L.; Knox, C.M.; Moore, S.D.; Hill, M.P.; Szewczyk, B. Morphological, genetic and biological characterisation of a novel alphabaculovirus isolated from Cryptophlebia peltastica (Lepidoptera: Tortricidae). J. Invertebr. Pathol. 2018, 157, 90-99, doi:10.1016/j.jip.**2018**.08.006.

32. Ma, X.-C.; Shang, J.-Y.; Yang, Z.-N.; Bao, Y.-Y.; Xiao, Q.; Zhang, C.-X. Genome sequence and organization of a nucleopolyhedrovirus that infects the tea looper caterpillar, Ectropis obliqua. Virology **2007**, 360, 235-246, doi:10.1016/j.virol.2006.10.024.

33. Tang, X.-D.; Xiao, Q.; Ma, X.-C.; Zhu, Z.-R.; Zhang, C.-X. Morphology and genome of Euproctis pseudoconspersa nucleopolyhedrovirus. Virus Genes **2009**, 38, 495-506, doi:10.1007/s11262-009-0355-0.

34. Noune, C.; Hauxwell, C. Complete Genome Sequences of Helicoverpa armigera single nucleopolyhedrovirus Strains AC53 and H25EA1 from Australia. Genome Announc **2015**, 3, e01083-01015, doi:10.1128/genomeA.01083-15.

35. Tang P, Z.H., Li Y, Han B, Wang G, Qin Q, et al. Genomic sequencing and analyses of HearMNPV--a new Multinucleocapsid nucleopolyhedrovirus isolated from Helicoverpa armigera. Virology journal **2012**, 9, doi:10.1186/1743-422X-9-168.

36. Chen X, W.I., Tarchini R, Sun X, Sandbrink H, et al. The sequence of the Helicoverpa armigera single nucleocapsid nucleopolyhedrovirus genome. J. Gen. Virol. **2001**, 82, 241-257.

37. Zhang, C.X.; Ma, X.C.; Guo, Z.J. Comparison of the complete genome sequence between C1 and G4 isolates of the Helicoverpa armigera single nucleocapsid nucleopolyhedrovirus. Virology **2005**, 333, 190-199, doi:10.1016/j.virol.2004.12.028.

38. Ogembo JG, C.B., Shikata M, Chaeychomsri S, Kobayashi M, et al. Comparative genomic sequence analysis of novel Helicoverpa armigera nucleopolyhedrovirus (NPV) isolated from Kenya and three other previously sequenced Helicoverpa spp. NPVs. Virus Genes **2009**, 39, 261-272, doi:10.1007/s11262-009-0389-3).

39. Chen X, Z.W., Wong J, Chun G, Lu A, et al. Comparative analysis of the complete genome sequences of Helicoverpa zea and Helicoverpa armigera single-nucleocapsid nucleopolyhedroviruses. J. Gen. Virol. **2002**, 83, 673-684.

40. Rohrmann, G.F.; Erlandson, M.A.; Theilmann, D.A. The genome of a baculovirus isolated from Hemileuca sp. encodes a serpin ortholog. Virus Genes **2013**, 47, 357-364, doi:10.1007/s11262-013-0951-x.

41. Nguyen, T.T.; Suryamohan, K.; Kuriakose, B.; Janakiraman, V.; Reichelt, M.; Chaudhuri, S.;

Guillory, J.; Divakaran, N.; Rabins, P.E.; Goel, R., et al. Comprehensive analysis of single molecule sequencing-derived complete genome and whole transcriptome of Hyposidra talaca nuclear polyhedrosis virus. Sci. Rep. **2018**, 8, 8924-8935, doi:10.1038/s41598-018-27084-y.

42. Rohrmann, G.F.; Erlandson, M.A.; Theilmann, D.A. Genome Sequence of an Alphabaculovirus Isolated from the Oak Looper, Lambdina fiscellaria, Contains a Putative 2-Kilobase-Pair Transposable Element Encoding a Transposase and a FLYWCH Domain-Containing Protein. Genome Announc **2015**, 3, e00186-00115, doi:10.1128/genomeA.00186-15.

43. Xiao, H.; Qi, Y. Genome sequence of Leucania seperata nucleopolyhedrovirus. Virus Genes **2007**, 35, 845-856, doi:10.1007/s11262-007-0106-z.

44. 44. Kuzio J, P.M., Harwood SH, Funk CJ, Evans JT, et al. Sequence and analysis of the genome of a baculovirus pathogenic for Lymantria dispar. Virology **1999**, 253, 17-34.

45. Nai, Y.S.; Wu, C.Y.; Wang, T.C.; Chen, Y.R.; Lau, W.H.; Lo, C.F.; Tsai, M.F.; Wang, C.H. Genomic sequencing and analyses of Lymantria xylina multiple nucleopolyhedrovirus. BMC Genomics **2010**, 11, 116-130, doi:10.1186/1471-2164-11-116.

46. Gencer, D.; Nalcacioglu, R.; Demirbag, Z.; Demir, I. Complete genome sequence analysis of the Malacosoma neustria nucleopolyhedrovirus from Turkey. Virus Genes **2018**, 54, 706-718, doi:10.1007/s11262-018-1595-7.

47. Choi, J.B.; Heo, W.I.; Shin, T.Y.; Bae, S.M.; Kim, W.J.; Kim, J.I.; Kwon, M.; Choi, J.Y.; Je, Y.H.; Jin, B.R., et al. Complete genomic sequences and comparative analysis of Mamestra brassicae nucleopolyhedrovirus isolated in Korea. Virus Genes **2013**, 47, 133-151, doi:10.1007/s11262-013-0922-2.

48. Li, Q.; Donly, C.; Li, L.; Willis, L.G.; Theilmann, D.A.; Erlandson, M. Sequence and organization of the Mamestra configurata nucleopolyhedrovirus genome. Virology **2002**, 294, 106-121, doi:10.1006/viro.2001.1313.

49. Li, L.; Li, Q.; Willis, L.G.; Erlandson, M.; Theilmann, D.A.; Donly, C. Complete comparative genomic analysis of two field isolates of Mamestra configurata nucleopolyhedrovirus-A. J. Gen. Virol. **2005**, 86, 91-105, doi:10.1099/vir.0.80488-0.

50. Harrison, R.L.; Mowery, J.D.; Rowley, D.L.; Bauchan, G.R.; Theilmann, D.A.; Rohrmann, G.F.; Erlandson, M.A. The complete genome sequence of a third distinct baculovirus isolated from the true armyworm, Mythimna unipuncta, contains two copies of the lef-7 gene. Virus Genes **2017**, 54, 297-310, doi:10.1007/s11262-017-1525-0.

51. Thumbi, D.K.; Eveleigh, R.J.; Lucarotti, C.J.; Lapointe, R.; Graham, R.I.; Pavlik, L.; Lauzon, H.A.; Arif, B.M. Complete sequence, analysis and organization of the Orgyia leucostigma nucleopolyhedrovirus genome. Viruses **2011**, 3, 2301-2327, doi:10.3390/v3112301.

52. Harrison RL; Rowley DL; Mowery JD; Bauchan GR; Burand JP. The Operophtera brumata Nucleopolyhedrovirus (OpbuNPV) Represents an Early, Divergent Lineage within Genus Alphabaculovirus. *Viruses* **2017,** 9(10):307, doi:10.3390/v9100307

53. Rohrmann, G.F.; Erlandson, M.A.; Theilmann, D.A. A Distinct Group II Alphabaculovirus Isolated from a Peridroma Species. Genome Announc **2015**, 3, e00185-00115, doi:10.1128/genomeA.00185-15.

54. Ardisson-Araujo, D.M.; Lima, R.N.; Melo, F.L.; Clem, R.J.; Huang, N.; Bao, S.N.; Sosa-Gomez, D.R.; Ribeiro, B.M. Genome sequence of Perigonia lusca single nucleopolyhedrovirus: insights into the evolution of a nucleotide metabolism enzyme in the family baculoviridae. Sci. Rep. **2016**, 6, 24612-24626, doi:10.1038/srep24612.

55. Craveiro, S.R.; Inglis, P.W.; Togawa, R.C.; Grynberg, P.; Melo, F.L.; Ribeiro, Z.M.; Ribeiro, B.M.; Bao, S.N.; Castro, M.E. The genome sequence of Pseudoplusia includens single nucleopolyhedrovirus and an analysis of p26 gene evolution in the baculoviruses. BMC Genomics **2015**, 16, 127, doi:10.1186/s12864-015-1323-9.

56. WF IJ, v.S.E., Heldens JG, Broer R, Zuidema D, et al. Sequence and organization of the Spodoptera exigua multicapsid nucleopolyhedrovirus genome. J. Gen. Virol. **1999**, 80, 3289-3304.

57. Harrison, R.L.; Puttler, B.; Popham, H.J. Genomic sequence analysis of a fast-killing isolate of Spodoptera frugiperda multiple nucleopolyhedrovirus. J. Gen. Virol. **2008**, 89, 775-790, doi:10.1099/vir.0.83566-0.

58. Jonathan E. B., El-Sayed A. E., Robert L. Harrison, Daniel L. Rowley, Michael E. S., Dawn E. Gundersen-Rindal, Holly J.R. Popham. Determination and analysis of the genome sequence of Spodoptera littoralis multiple nucleopolyhedrovirus. Virus. Research. **2013**, 171,1, 194-208.

59. Pang, Y.; Yu, J.; Wang, L.; Hu, X.; Bao, W.; Li, G.; Chen, C.; Han, H.; Hu, S.; Yang, H. Sequence analysis of the Spodoptera litura multicapsid nucleopolyhedrovirus genome. Virology **2001**, 287, 391-404, doi:10.1006/viro.2001.1056.

60. Liu, X.; Yin, F.; Zhu, Z.; Hou, D.; Wang, J.; Zhang, L.; Wang, M.; Wang, H.; Hu, Z.; Deng, F. Genomic sequencing and analysis of Sucra jujuba nucleopolyhedrovirus. PLoS One **2014**, 9,

e110023, doi:10.1371/journal.pone.0110023.

61. Willis, L.G.; Seipp, R.; Stewart, T.M.; Erlandson, M.A.; Theilmann, D.A. Sequence analysis of the complete genome of Trichoplusia ni single nucleopolyhedrovirus and the identification of a baculoviral photolyase gene. Virology **2005**, 338, 209-226, doi:10.1016/j.virol.2005.04.041.

62. Wormleaton, S.; Kuzio, J.; Winstanley, D. The complete sequence of the Adoxophyes orana granulovirus genome. Virology **2003**, 311, 350-365, doi:10.1016/s0042-6822(03)00149-1.

63. Escasa, S.R.; M., L.H.A.; Mathur, A.C.; Krell, P.J.; Arif, B. Sequence analysis of the Choristoneura occidentalis granulovirus genome. J. Gen. Virol. **2006**, 87, 1917-1933, doi:10.1099/vir.0.81792-0.

64. Liang, Z.; Zhang, X.; Yin, X.; Cao, S.; Xu, F. Genomic sequencing and analysis of Clostera anachoreta granulovirus. Arch. Virol. **2011**, 156, 1185-1198, doi:10.1007/s00705-011-0977-0.

65. Liang, Z; Zhang, X.; Yin, X.; Song, X.; Shao, X.; Wang, L.. Comparative analysis of the genomes of Clostera anastomosis (L.) granulovirus and Clostera anachoreta granulovirus. Archives of Virology **2013**, 158, 2109-2114, doi:10.1007/s00705-013-1710-y.

66. Yin, F.; Zhu, Z.; Liu, X.; Hou, D.; Wang, J.; Zhang, L.; Wang, M.; Kou, Z.; Wang, H.; Deng, F., et al. The Complete Genome of a new betabaculovirus from Clostera anastomosis. PLoS One **2015**, 10, e0132792, doi:10.1371/journal.pone.0132792.

67. Han, G.; Xu, J.; Liu, Q.; Li, C.; Xu, H.; Lu, Z. Genome of Cnaphalocrocis medinalis Granulovirus, the first Crambidae-Infecting betabaculovirus isolated from Rice Leaffolder to sequenced. PLoS One **2016**, 11, e0147882, doi:10.1371/journal.pone.0147882.

68. Luque T, F.R., Crook N, O'Reilly DR, Winstanley D. The complete sequence of the Cydia pomonella granulovirus genome. J. Gen. Virol. **2001**, 82, 2531-2547.

69. Lange, M.; Jehle, J.A. The genome of the Cryptophlebia leucotreta granulovirus. Virology **2003**, 317, 220-236, doi:10.1016/s0042-6822(03)00515-4.

70. Ardisson-Araujo, D.M.; Melo, F.L.; Clem, R.J.; Wolff, J.L.; Ribeiro, B.M. A Betabaculovirus-Encoded gp64 Homolog Codes for a Functional Envelope Fusion Protein. J. Virol. **2016**, 90, 1668-1672, doi:10.1128/JVI.02491-15.

71. Ferrelli ML, S.R., Biedma ME, Berretta MF, Haase S, et al. Genome of Epinotia aporema granulovirus (EpapGV), a polyorganotropic fast killing betabaculovirus with a novel

thymidylate kinase gene. BMC Genomics **2012**, 13, 548-562.

72. Ardisson-Araújo DMP, d.M.F., Andrade MdS, Sihler W, Báo SN, Ribeiro BM, et al. Genome sequence of Erinnyis ello granulovirus (ErelGV), a natural cassava hornworm pesticide and the first sequenced sphingid-infecting betabaculovirus. BMC Genomics **2014**, 15, 856, doi:10.1186/1471-2164-15-856.

73. Harrison, R.L.; Popham, H.J. Genomic sequence analysis of a granulovirus isolated from the Old World bollworm, Helicoverpa armigera. Virus Genes **2008**, 36, 565-581, doi:10.1007/s11262-008-0218-0.

74. Harrison, R.L.; Rowley, D.L.; Mowery, J.; Bauchan, G.R.; Theilmann, D.A.; Rohrmann, G.F.; Erlandson, M.A. The complete genome sequence of a second distinct betabaculovirus from the True Armyworm, Mythimna unipuncta. PLoS One **2017**, 12, e0170510, doi:10.1371/journal.pone.0170510.

75. Taha A; Nour-El-Din A,; Croizier L; Ferber ML; Croizier G. Comparative analysis of the granulin regions of the Phthorimaea operculella and Spodoptera littoralis granuloviruses. Virus Genes. **2000,** 10;21(3):147-55.

76. Hashimoto, Y.; Hayakawa, T.; Ueno, Y.; Fujita, T.; Sano, Y.; Matsumoto, T. Sequence analysis of the Plutella xylostella granulovirus genome. Virology **2000**, 275, 358-372, doi:10.1006/viro.2000.0530.

77. Zhang, B.Q.; Cheng, R.L.; Wang, X.F.; Zhang, C.X. The Genome of Pieris rapae Granulovirus. J. Virol. **2012**, 86, 9544, doi:10.1128/JVI.01431-12.

78. Cuartas, P.E.; Barrera, G.P.; Belaich, M.N.; Barreto, E.; Ghiringhelli, P.D.; Villamizar, L.F. The complete sequence of the first Spodoptera frugiperda Betabaculovirus genome: a natural multiple recombinant virus. Viruses **2015**, 7, 394-421, doi:10.3390/v7010394.

79. Wang, Y.; Choi, J.Y.; Roh, J.Y.; Liu, Q.; Tao, X.Y.; Park, J.B.; Kim, J.S.; Je, Y.H. Genomic sequence analysis of granulovirus isolated from the tobacco cutworm, Spodoptera litura. PLoS One **2011**, 6, e28163, doi:10.1371/journal.pone.0028163.

80. Hayakawa T, K.R., Okano K, Seong SI, Goto C, et al. Sequence analysis of the Xestia c-nigrum granulovirus genome. Virology **1999**, 262, 277-297.

81. Duffy, S.P.; Young, A.M.; Morin, B.; Lucarotti, C.J.; Koop, B.F.; Levin, D.B. Sequence analysis and organization of the Neodiprion abietis nucleopolyhedrovirus genome. J. Virol. **2006**, 80, 6952-6963, doi:10.1128/JVI.00187-06.

82. Lauzon, H.A.; Lucarotti, C.J.; Krell, P.J.; Feng, Q.; Retnakaran, A.; Arif, B.M. Sequence and organization of the Neodiprion lecontei nucleopolyhedrovirus genome. J. Virol. **2004**, 78, 7023-7035, doi:10.1128/JVI.78.13.7023-7035.2004.

83. Garcia-Maruniak, A.; Maruniak, J.E.; Zanotto, P.M.; Doumbouya, A.E.; Liu, J.C.; Merritt, T.M.; Lanoie, J.S. Sequence analysis of the genome of the Neodiprion sertifer nucleopolyhedrovirus. J. Virol. **2004**, 78, 7036-7051, doi:10.1128/JVI.78.13.7036-7051.2004.

84. Afonso, C.L.; Tulman, E.R.; Lu, Z.; Balinsky, C.A.; Moser, B.A.; Becnel, J.J.; Rock, D.L.; Kutish, G.F. Genome sequence of a baculovirus pathogenic for Culex nigripalpus. J. Virol. **2001**, 75, 11157-11165.
